# Supplementary material for: Replication Activities of Major 5′ Terminally Deleted Group-B Coxsackievirus RNA Forms Decrease PCSK2 mRNA Expression Impairing Insulin Maturation in Pancreatic Beta Cells
Source: Viruses. 2022 Dec 13;14(12):2781. doi: 10.3390/v14122781 (PMC9788552; doi:10.3390/v14122781)
Supplement: Supplementary file 1 [file viruses-14-02781-s001.zip › viruses-1990784-supplementary.pdf]

Supplementary material

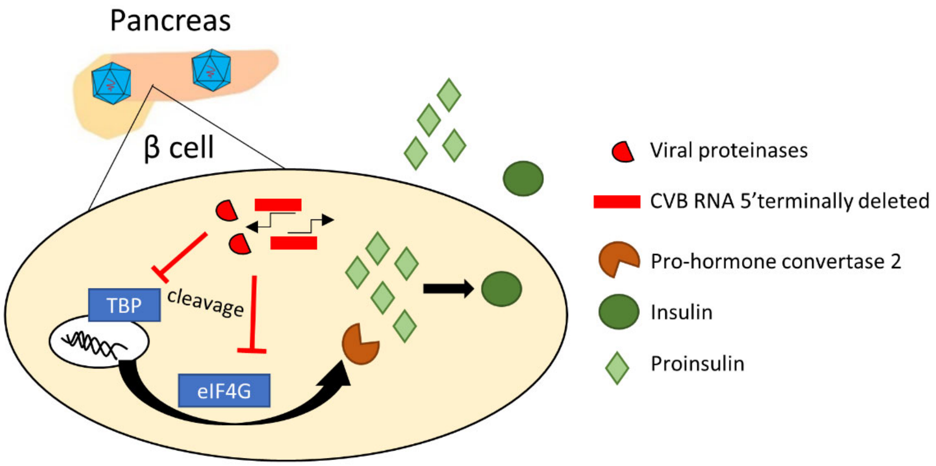

Figure S1 : Schematic representation of the scientific hypothesis. Replication activities of CVB-TD RNA forms produced low levels of viral proteins, sufficient to cleave host cells factors such as TBP or eIF4G. These viral proteinase cleavage activities could result in a decrease in PCSK2 enzymatic activity, with the accumulation of proinsulin and a decrease in insulin secretion.

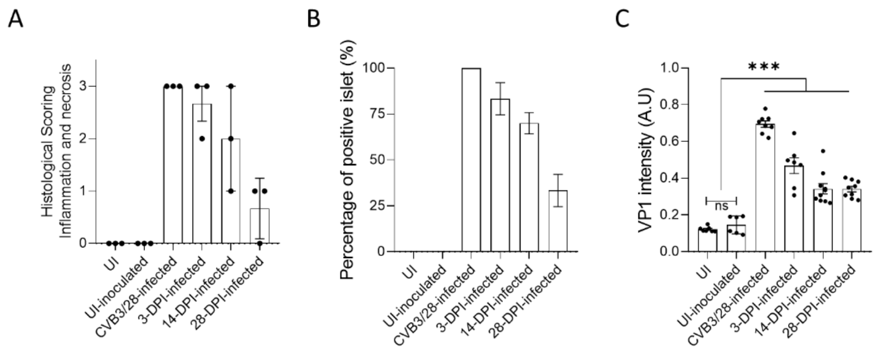

Figure S2: Histological scoring and Viral Protein 1 positivity in pancreas of DBA/2J mice at 7 days post infection. (A) Histological scoring for inflammation and necrosis in HES stained slices of pancreas, from uninfected or infected DBA/2J mice, or inoculated with homogenized pancreas from 3-DPI, 14-DPI or 28-DPI and uninfected mice (n=3). (B) Percentages of islets positive for VP1 in immunohistochemistry (n=3). (C) VP1 intensity on slices of pancreas (n=3, with two to three ROI per slice). Data presented as mean +/- SEM. Mann-Whitney test: ***= p<0.001. ns: non-significant. ROI: Region of interest. VP1: viral protein 1. UI: uninfected. CVB3: coxsackievirus B3. AU: arbitrary unit. DPI: days post infection.

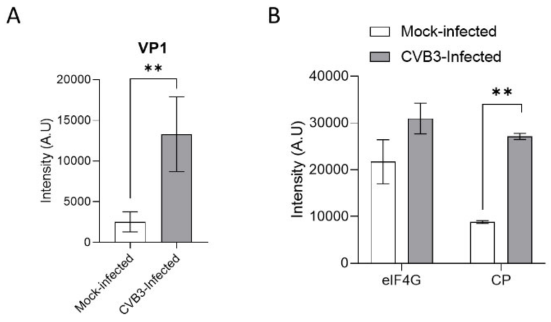

Figure S3: Western blot intensity analyses. (A) Viral protein 1 intensity after 24 hours of incubation (mock-infected) or CVB3/28 infection, at a Multiplicity of Infection of 1 (n=3). (B) eIF4G and its cleavage product intensities after 24 hours of incubation (mock-infected) or CVB3/28 infection, at a Multiplicity of Infection of 1 (n=2). Data presented as mean +/- SD. Mann-Whitney test: **= $p<0.01$. VP1: viral protein 1. UI: uninfected. CVB3: Coxsackievirus B3. AU: arbitrary unit. CP: cleavage product. eIF4G: Eukaryotic translation initiation factor 4 G.
